# Long-term quality of life in children after balloon dilatation for subglottic and tracheal stenosis: Eight years’ experience

**DOI:** 10.1097/MD.0000000000044428

**Published:** 2025-09-05

**Authors:** Masoumeh Ghasempour Alamdari, Mohammad Reza Modaresi, Rohola Shirzadi, Saminnaz Kazemi, Seyed Hossein Mirlohi

**Affiliations:** a Department of Pediatric Pulmonology, Tehran University of Medical Sciences, Bahrami Hospital, School of Medicine, Tehran, Iran; b Pediatric Respiratory Disease and Sleep Medicine Research Center, Tehran University of Medical Sciences, Tehran, Iran; c Pediatric Respiratory Disease and Sleep Medicine Research Center, Tehran University of Medical Sciences, Tehran, Iran; d Tehran University of Medical Sciences, Tehran, Iran; e Pediatric Respiratory Disease and Sleep Medicine Research Center, Tehran University of Medical Sciences, Tehran, Iran.

**Keywords:** balloon dilatation, pediatric, pediatric subglottic stenosis, quality of life, subglottic stenosis

## Abstract

Subglottic stenosis (SGS) is a significant cause of breathing obstruction in pediatric patients, predominantly acquired due to prolonged endotracheal intubation. The primary aim of this study was to evaluate long-term quality of life in children after Balloon Dilatation for subglottic and tracheal stenosis. This cross-sectional study evaluated pediatric patients with SGS or tracheal stenosis treated with balloon dilatation at a children’s medical center in Tehran, Iran, from 2014 to 2021. The study included patients aged 1 month to 15 years, excluding those with other tracheal conditions, congenital SGS, previous interventions for SGS, or significant comorbidities. Data collected included demographics, dyspnea grading, balloon dilatation frequency, hospital stay duration, and Pediatric Quality of Life Inventory (PedsQL) scores. Fifteen patients (12 male, 3 female) with a mean age of 7.27 ± 1.53 years were included. Most patients had a history of intubation (86.7%) and exhibited grade I or II dyspnea. The average hospital stay post-procedure was 2.2 ± 1.1 days. PedsQL scores indicated high quality of life in physical, psychosocial, social, and emotional domains for most patients. A significant association was found between balloon dilatation frequency and social functioning (*P* = .02), but not with other PedsQL aspects or intubation history. Balloon dilatation is an effective, minimally invasive treatment for pediatric SGS, improving clinical outcomes and quality of life. Further studies with larger sample sizes and prospective designs are recommended to confirm these findings and assess long-term outcomes.

## 1. Introduction

Subglottic stenosis (SGS) is a frequent cause of breathing obstruction in pediatric cases. It can be present at birth (congenital) or develop later in life (acquired). In contrast to the uncommon congenital form arising from improper relining of the laryngeal passage, acquired SGS, representing nearly 90% of cases, can stem from various factors.^[1,2]^ The most frequent culprit is extended use of a breathing tube (endotracheal intubation).^[3,4]^ Difficulty catching breath, labored breathing (dyspnea), and a high-pitched whistling sound during inhalation are the most frequent signs experienced by patients. Studies suggest that following endotracheal intubation, the development of acquired SGS occurs in approximately 2% of neonates and 11% of children under the age of 5.^[5]^

Therapeutic interventions for subglottic stenosis encompass both endoscopic and open surgical approaches. Endoscopic techniques include laser or cold knife incision, bouginage, and balloon dilatation. Open surgical options comprise anterior cricoid split, laryngotracheoplasty, laryngotracheal resection, and cricotracheal resection.^[6]^ The initial application of balloon technology in treating subglottic stenosis involved intra-arterial balloons during the 1980s. This approach leveraged radial force to dilate the stenotic segment, offering a more precise and controlled method compared to previous techniques. By distributing force evenly, the risk of tissue damage and subsequent scar formation was mitigated, potentially reducing the likelihood of restenosis.^[7]^ Endoscopic balloon dilatation (EBD) offers a less invasive alternative to traditional surgical interventions. By precisely applying radial pressure, EBD minimizes tissue trauma, potentially reducing the risk of complications associated with surgical approaches. This technique has demonstrated efficacy across various medical specialties, including the management of vascular and esophageal strictures. While its application in airway management is relatively newer, the potential benefits of reduced invasiveness and targeted treatment make it a promising option for subglottic stenosis.^[8,9]^

Studies have shown promising results for EBD in treating acquired SGS. One investigation reported a success rate of 82.3% for patients with various severities, including those with severe stenosis and elongated lesions.^[10,11]^ Another study achieved an even higher success rate of 86% using EBD for acquired SGS.^[12]^ However, after EBD, diligent postoperative monitoring is essential to identify and promptly address any airway complications, which can potentially be life-threatening. The development of edema at the surgical site may exacerbate airway obstruction, necessitating vigilant observation and prompt intervention. Also, one of the challengeable things about using this method is related to its long-term effectiveness and its effect of patient’s quality of life.^[6,13]^ In fact, most of the studies have evaluated the short-term outcomes of this method, and the long-term outcomes have not been investigated.

In the hospitals of Iran, as a country where the prevalence of pediatric SGS is high, the process of treating this disease as well as long-term effect of EBD has become a challenge for specialists. Therefore, the purpose of the present study was to evaluate the effectiveness of balloon dilatation in comparison with other treatment methods in pediatric patients with SGS.

## 2. Materials and methods

### 2.1. Study subjects

This study was a cross-sectional study that was conducted among the pediatric patients suffered from subglottic stenosis and tracheal stenosis admitted to children’s medical center in Tehran, Iran and treated with balloon dilatation between 2014 and 2021. We included patients with age range of 1 month to 15 years old. Patients were excluded if they had other coexisting tracheal pathologic conditions, congenital SGS, previous intervention for their SGS, or comorbidities requiring a high likelihood of prolonged ventilation in the future. The protocol of this project was reviewed and approved by the ethics committee of Tehran University of Medical Sciences (IR.TUMS.CHMC.REC.1400.072). Due to the rarity of SGS and the specific inclusion criteria (patients treated with balloon dilatation between 2014 and 2021, aged 1 month to 15 years, excluding those with congenital SGS or significant comorbidities), a total of 15 patients were eligible for inclusion. A power analysis was not conducted due to the retrospective nature of the study and the limited patient population at our center. However, this sample size is consistent with similar studies on pediatric SGS, where small cohorts are common due to the condition’s low prevalence.^[5,7]^

### 2.2. Surgical technique

The present study was conducted in a descriptive and retrospective manner based on the medical records of the patients. However, in this hospital, the balloon procedure performed as follows. The balloon dilation treatment program consisted of a sequence of 1 to 4 dilation procedures; each performed within a maximum timeframe of 6 months. The dilation procedure utilized a high-pressure balloon catheter inserted endoscopically. This minimally invasive technique was performed under general anesthesia, allowing patients to breathe naturally following a standardized protocol. To visualize the stenosis and guide the procedure, a small, rigid scope was inserted through the mouth or nose under general anesthesia. A specialized balloon catheter was then introduced through the scope and positioned within the narrowed airway. The balloon was then inflated using a specialized device for precise pressure control. Inflation continued for 30 seconds or until the patient’s oxygen level dipped below 92%. The balloon size was chosen based on the estimated ideal diameter of the healthy cricoid cartilage. The smallest balloon used had a diameter of 6 millimeters. Each session under general anesthesia could involve repeating this dilation process 2 or 3 times. Additionally, when available, a topical medication was applied to the area for 1 to 2 minutes after the procedure.

### 2.3. Data collection

First, we searched the Hospital Records Database and all the patients’ medical records that met the inclusion criteria were selected. Then, according to the information in the files, the checklists for each patient were completed. In the absence of sufficient information, the case was excluded from the study. In other words, in this cross-sectional study, the patients’ medical records with subglottic stenosis and tracheal stenosis, who admitted to pediatric medical center between 2014 and 2021 and underwent balloon dilatation, were evaluated.

First, demographic variables such as age, gender, birth weight, child’s growth level, routine pregnancy screening, number of children in the family, having seasonal allergies, being a passive smoker were recorded from the patients’ files. The growth rate was defined as normal growth and poor growth based on the report provided by the local health centers. Then, the basic information of the disease, including the grading of dyspnea, the number of balloon dilatation times, the number of days the patients were hospitalized after the balloon dilatation, and the history of intubation were also collected and recorded. Grading of dyspnea was also measured based on the Modified Medical Research Council (mMRC) scale.^[14]^

The mMRC dyspnea scale is a tool used to measure how much shortness of breath limits a person’s daily activities. This scale is a rating tool to measure the degree of disability caused by shortness of breath in daily activities on a scale from 0 to 4. The grading system of this tool is as follows: Grade zero: no shortness of breath except during intense sports, Grade 1: Shortness of breath when walking hurriedly on the surface or walking down a small hill, Grade 2: walks slower than people of the same age on the surface or has shortness of breath when walking at her/his own speed on the surface and also has to stop to breathe, Grade 3: after walking ~100 m or after a few minutes on the surface, stops for breath, Grade 4: Too breathless to leave the house or breathless when dressing or undressing.

Next, by contacting the patients, questions were asked about the quality of life of children after balloon dilatation at least 2 years after balloon dilatation surgery. For this purpose, the pediatric quality of life (PedsQL) Inventory Generic Core Scales was used to measure the level of quality of life. The PedsQL generic core scales is a multidimensional instrument designed to measure health-related quality of life (HRQOL) in children and adolescents aged 2 to 18 years. It is a patient-reported or parent proxy-reported tool, meaning children can answer the questions themselves (depending on age and development) or a parent can answer on their behalf. Potential study participants were identified from the patient population. Subsequently, parents of these children were approached by a research nurse for informed consent prior to their scheduled medical consultation. Prior to study participation, written informed consent was obtained from parents or legal guardians. Additionally, age-appropriate children provided assent to participate. Data collection involved separate completion of the PedsQL questionnaires by parents and children. To facilitate questionnaire completion and address any participant queries, a research nurse was available to provide assistance as needed. For younger children, the research nurse administered the PedsQL questionnaire. This study relied on quantitative PedsQL scores and did not collect qualitative data, such as interviews or open-ended surveys, from patients or parents to explore their experiences or satisfaction with the treatment. Future studies could incorporate such qualitative methods to provide deeper insights into patient and family perspectives.

The PedsQL generic core scales are considered “generic” because they assess core aspects of HRQOL that are applicable to a wide range of health conditions. It’s “modular” as it can be used independently or integrated with disease-specific modules from the PedsQL measurement system. The different items of this questionnaire include different areas of physical, psychosocial, social, emotional and school functions.

The scoring method in this scale is reversed and from zero to 100, so that higher scores indicate better HRQOL. In order to interpret the scores of all the items of each function, we add up and divide by the number of items, and the score of each function is determined from zero to 100 (in the other words, the average is calculated as the sum of the items on the number of answered items). Then Based on the ranking of this score, if this number is between 75 and 100, it is defined as good performance, between 25 and 75 as average performance, and below 25 as poor performance.

### 2.4. Statistical analysis

All of data were analyzed by SPSS version 20 (Chicago). Frequency was reported as ratio (percentage) for qualitative variables and as mean ± SD for quantitative variables. For data analysis, univariate analysis methods such as independent T test and chi square test were used depending on the type of variable. *P* < .05 was considered statistically significant.

## 3. Results

The baseline characteristics of patients was summarized in Table 1. Fifteen patients were included in this study (12 male and 3 female). The mean age of the participants was 7.27 ± 1.53 years old. Seven of the patients had delayed growth and the average birth weight of the patients was 2.77 ± 0.55 kg. Also, the mothers of 2 patients had not performed prepregnancy screenings. Moreover, 2 of the patients had allergies and 5 were passive smokers.

**Table 1:** Demographic characteristic of patients.

Variables	Patients (N = 15)
Age (year)	7.27 ± 1.53
Sex, male (%)	12 (80%)
Allergy (%)	2 (13.3%)
Mothers without pregnancy screening	2 (13.3%)
Passive smoker	5 (33.3%)
Dyspnea (grade 1)	11 (73.3%)
Dyspnea (grade 2)	4 (26.7%)
History of intubation	13 (86.7%)
Frequency of dilation	1 to 4
Stay at the hospital	2.2 ± 1.1

In term of dyspnea severity, 4 patients had grade II of dyspnea and the rest of them suffered from grade I. Also, 13 patients (86.7%) had history of intubation and in term of frequency of balloon dilatation, it varied between at least once and at most 4 times. Finally, the average number of stay at the hospital of patients after balloon dilatation was 2.2 ± 1.1 days.

**Figure 1.**
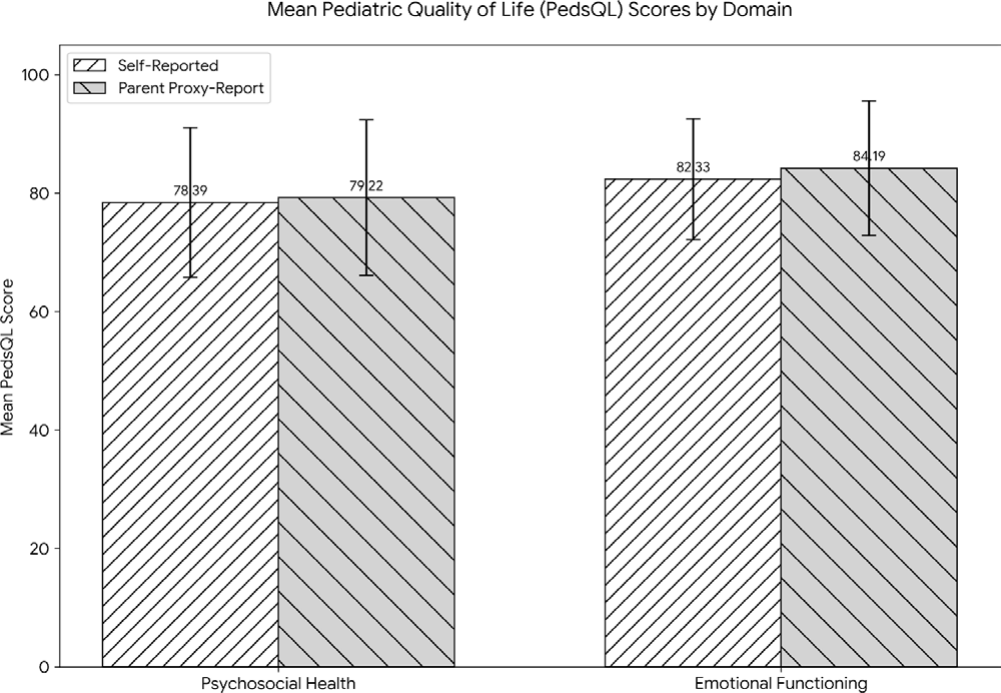
Mean PedsQL score by domain. PedsQL = pediatric quality of life.

The means and standard deviations for the PedsQL is presented in Table 2. The average total self-reported PedsQL score was 78.39 ± 12.63 and 79.22 ± 13.15 for proxy-report. Also, in term of different functions in the self-reported items, the highest score belonged to emotional functioning (82.33 ± 10.23). Similar to self-reported PedsQL, in the reports of the parents, the emotional functioning got the highest score (84.19 ± 11.39) (Fig. 1).

**Table 2:** Scale descriptives for PedsQL generic core scales child self-report and parent proxy-report.

Scale	N	Mean	SD
Self-report			
Total score	15	78.39	12.63
Physical health	15	81.32	12.18
Psychosocial health	15	74.93	13.65
Emotional functioning	15	82.33	10.23
Social functioning	15	81.66	12.9
School functioning	11	73.06	9.26
Proxy-report			
Total score	15	79.22	13.15
Physical health	15	82.56	14.03
Psychosocial health	15	75.36	12.44
Emotional functioning	15	84.19	11.39
Social functioning	15	81.75	12.21
School functioning	11	75.19	10.92

PedsQL = pediatric quality of life, SD = standard deviation.

Also, as shown in Table 3, in term of physical function and social functioning of PedsQL, 11 patients experienced high quality of life and 4 patients experienced moderate quality of life. Also, in term of emotional functioning, 14 patients experienced high quality of life and 1 patient experienced moderate quality of life. Moreover, we found a high quality of life in term of psychosocial health (80%) and school functioning (72.2%).

**Table 3:** Classification of patients based on the quality of life score.

	Number of patients	Percentage%
Physical health		
High	11	73.3
Moderate	4	26.7
Poor	0	0
Psychosocial health		
High	12	80
Moderate	3	20
Poor	0	0
Emotional functioning		
High	14	93.3
Moderate	1	6.7
Poor	0	0
School functioning		
High	8	72.3
Moderate	3	27.7
Poor	0	0
Social functioning		
High	11	73.3
Moderate	4	26.7
Poor	0	0

Finally, we found a significant association between frequency of balloon dilatation and social functioning of PedsQL scale (*P* = .02). However, we couldn’t find any association between frequency of balloon dilatation with history of intubation and other aspects of PedsQL scale (*P* > .05). Visual aids, such as graphs or charts depicting PedsQL scores or images of the balloon dilatation procedure, were not included in this study. Such aids could enhance the presentation and interpretation of the results and methodology in future reports.

## 4. Discussion

This cross-sectional study investigated the effectiveness of balloon dilatation on long-term quality of life in children after balloon dilatation for SGS in children’s medical center in Tehran, Iran. The results of the present study showed that this method can improve the quality of life of patients in the long term in the total score as well as different aspects of quality of life.

Our study aligns with previous research highlighting the efficacy of balloon dilatation as a minimally invasive treatment modality. Erlandsson et al in a cross-sectional study among 40 patients after balloon dilatation evaluated the efficacy of this method on quality of life using dyspnea index and short form health survey‑36. They found that patients who received balloon dilatation experienced a significant improvement in dyspnea index and short form health survey‑36 scores.^[15]^ Unlike our study, this study was conducted on adults.

Patient-reported outcome measures such as PedsQL and dyspnea index offer a valuable perspective on surgical outcomes by directly capturing patients’ experiences.^[16]^ This approach mitigates potential biases inherent in clinician-reported assessments, allowing for a more accurate and patient-centered evaluation of treatment efficacy. By focusing on outcomes deemed important by patients themselves, patient-reported outcome measures enhance the relevance and applicability of research findings.^[17]^ The reliance on PedsQL scores in our study provided robust quantitative data on HRQOL but did not capture qualitative insights into patient and parent experiences, such as satisfaction with the treatment or emotional impacts beyond standardized scales. Incorporating qualitative methods, such as semi-structured interviews or open-ended surveys, could provide a more comprehensive understanding of the lived experiences of children and their families posttreatment, as suggested by studies emphasizing the value of mixed-methods approaches in pediatric research.^[18]^ To accurately differentiate SGS from other respiratory conditions presenting with similar symptoms, objective functional assessments have been the focus of several investigations.^[19]^ These assessments aim to identify reliable diagnostic markers and inform treatment decisions, such as the need for surgical intervention.

While balloon dilatation is a minimally invasive procedure with demonstrated efficacy, it is not without potential complications. Postoperative airway edema is a common concern, which may exacerbate airway obstruction and require vigilant monitoring or intervention.^[20]^ Restenosis, or recurrence of the stenosis, is another risk, with studies reporting recurrence rates ranging from 51.6% to 59.2% over follow-up periods.^[21]^ Rare but serious complications, such as airway rupture or mucosal injury, have also been reported, particularly in cases of severe stenosis or improper balloon sizing.^[8]^ Tuzuner et al (2022) noted that respiratory problems following EBD, including transient desaturation or laryngeal edema, were observed in some pediatric cases, emphasizing the need for careful patient selection and postoperative care.^[7]^ These risks highlight the importance of balancing the benefits of balloon dilatation – such as reduced surgical trauma and shorter hospital stays – with its potential complications, ensuring that clinicians are prepared to manage adverse events promptly.

Several studies have shown the superiority of this method over other methods in the treatment of. Zheng et al in their study among 49 pediatric patients with SGS compared the efficacy of balloon dilatation than open laryngotracheal reconstruction (OLR). The researchers employed a series of 1 to 7 dilatation procedures, with a reported success rate of 59.2%.^[22]^ In contrast, open surgical approaches, such as laryngotracheal reconstruction or cricotracheal resection, are often reserved for severe or complex SGS cases but carry higher risks of complications, including vocal cord dysfunction, prolonged recovery, and significant scarring.^[18]^ For instance, Narcy et al (1990) reported that OLR, while effective, can lead to postoperative complications in up to 20% of pediatric patients, including granulation tissue formation and airway compromise.^[18]^ CO2 laser excision, another alternative, has been compared directly with balloon dilatation. Erlandsson et al (2023) found no significant differences in treatment intervals between balloon dilatation and CO2 laser excision, suggesting comparable short-term efficacy. However, Ntouniadakis et al (2023) reported that balloon dilatation significantly reduced the risk of recurrence compared to CO2 laser excision, with a 3-year recurrence risk of 51.6% for balloon dilatation versus 73.7% for CO2 laser excision.^[23]^ These findings suggest that balloon dilatation may offer advantages in terms of lower recurrence rates and reduced invasiveness, though CO2 laser excision may be preferred in cases requiring precise tissue ablation. The choice of treatment modality depends on factors such as stenosis severity, patient age, and surgeon expertise, underscoring the need for individualized treatment plans.

The absence of a control group in our study limits the ability to directly compare balloon dilatation with other treatment modalities, such as OLR or CO2 laser excision. However, our findings are consistent with previous comparative studies, such as Zheng et al (2023),^[22]^ which reported a 59.2% success rate for balloon dilatation compared to OLR in pediatric SGS, and Ntouniadakis et al (2023),^[23,24]^ which found a lower recurrence rate with balloon dilatation (51.6%) compared to CO2 laser excision (73.7%). Future prospective studies incorporating a control group treated with alternative modalities are needed to establish the relative efficacy of balloon dilatation.

However, one of the big challenges is related to the long-term outcome and quality of life after ballon dilatation.^[9]^ The long-term evaluation of various functional aspects of the patient in relation to the quality of life with the report of the patients and their parents can be a suitable expression of the long-term success of these methods. Clinician decision-making regarding the management and treatment of SGS is primarily guided by the patient’s subjective experience of respiratory distress and its impact on functional capacity. Consistent with previous research, a notable discrepancy often exists between objectively measured airway obstruction and the subjective experience of dyspnea reported by patients with subglottic stenosis. This disparity underscores the importance of considering both physiological and patient-reported outcomes when managing this condition.^[25]^

While traditional OLR has demonstrated effectiveness in treating pediatric SGS, concerns persist regarding the potential for causing significant patient trauma during surgery.^[18,26]^ Advancements in endoscopic technology have positioned balloon dilatation as a potential alternative to traditional OLR for treating pediatric SGS. This minimally invasive approach utilizes a balloon catheter that applies radial pressure to the stenotic segment.^[27,28]^ Compared to OLR, which relies on instruments that exert shear force, balloon dilatation offers the potential to significantly reduce surgical trauma and minimize the risk of complications, such as airway rupture. Furthermore, the availability of smaller balloon diameters enhances the flexibility of the procedure, particularly for addressing severe stenosis of the airway where maneuverability within the narrowed passage is critical.^[22]^ Erlandsson et al in a retrospective analysis on medical records of patients with SGS compared the effectiveness of balloon dilatation and CO2 laser and they found that there weren’t significant differences in treatment intervals between these 2 models.^[20]^ However, in contrast with this study Ntouniadakis et al compared the effectiveness of balloon dilatation and CO2 laser excision in treating SGS in Sweden and it found that balloon dilatation significantly reduces the risk of recurrence compared to CO2 laser excision. Over a 3-year follow-up, the recurrence risk was 51.6% for balloon dilatation versus 73.7% for CO2 laser excision.^[23,29,30]^

While balloon dilatation has established efficacy in treating vascular diseases and esophageal stenosis, its application in pediatric airway management for SGS remains underutilized by some otolaryngologists. This cautious approach may be attributed to several factors, including a lack of long-term data on outcomes specifically in the airway compared to established surgical techniques, or potential concerns regarding the safety and efficacy of this relatively new approach in this delicate anatomical region.^[31,32]^ Despite the underutilization of balloon dilatation in pediatric airway management for SGS, several theoretical advantages make it a promising alternative to traditional rigid techniques. Unlike rigid instruments that exert concentrated shear forces, balloon dilatation utilizes a radial expansion principle. This allows for a more controlled and even distribution of pressure across the stenotic segment, minimizing the risk of trauma even in severe (pinpoint) stenosis. Consequently, balloon dilatation has the potential to reduce overall complication rates associated with airway intervention.^[33–37]^

The absence of a control group in our study limits the ability to directly compare the effectiveness of balloon dilatation with other treatment modalities, such as open surgical approaches like laryngotracheal reconstruction. This limitation is acknowledged, as the study focused on evaluating long-term quality of life outcomes in a specific cohort treated with balloon dilatation. Future research incorporating a control group treated with alternative modalities would provide a more robust comparison of treatment efficacy and long-term outcomes.

Despite the promising results, our study has several limitations. The sample size was relatively small (n = 15), which may limit the generalizability of our findings. This is primarily due to the low prevalence of pediatric SGS and the strict inclusion criteria, which excluded patients with congenital SGS, other tracheal pathologies, or prior interventions. While the sample size is comparable to other studies in this field,^[5,7,10]^ it may reduce the statistical power to detect associations between variables, such as the relationship between balloon dilatation frequency and other PedsQL domains. Additionally, the retrospective design of the study may introduce selection bias, and the reliance on medical records and parental reports for data collection could result in reporting biases. The absence of a control group treated with alternative methods, such as OLR, precludes definitive conclusions about the comparative effectiveness of balloon dilatation. Furthermore, the study did not incorporate qualitative data from interviews or surveys with patients and parents, which could have provided deeper insights into their experiences, satisfaction, and emotional impacts of the treatment. Future studies should aim to include a control group, larger sample sizes through multicenter collaboration, and qualitative data collection to strengthen the evidence base for balloon dilatation in pediatric SGS.

## 5. Conclusion

In conclusion, this study suggests that balloon dilatation is a promising, minimally invasive treatment option for pediatric patients with subglottic and tracheal stenosis, associated with high HRQOL scores across physical, emotional, social, psychosocial, and school functioning domains. The procedure’s safety profile and short hospital stays further support its potential as a primary treatment modality. However, the small sample size, retrospective design, and lack of a control group limit the ability to definitively establish its comparative effectiveness. Prospective, multicenter studies with larger sample sizes, control groups treated with alternative modalities (e.g., OLR or CO2 laser excision), and mixed-methods approaches incorporating qualitative data are needed to confirm these preliminary findings and optimize the application of balloon dilatation in pediatric SGS. Additionally, future studies should include visual aids, such as graphs or procedural images, to enhance the interpretability of results.

## Author contributions

**Conceptualization:** Masoumeh Ghasempour Alamdari, Rohola Shirzadi, Seyed Hossein Mirlohi.

**Data curation:** Masoumeh Ghasempour Alamdari, Rohola Shirzadi.

**Investigation:** Masoumeh Ghasempour Alamdari, Mohammad Reza Modaresi.

**Methodology:** Saminnaz Kazemi.

**Software:** Mohammad Reza Modaresi.

**Supervision:** Seyed Hossein Mirlohi.

**Validation:** Mohammad Reza Modaresi, Saminnaz Kazemi.

**Writing – original draft:** Rohola Shirzadi, Saminnaz Kazemi, Seyed Hossein Mirlohi.

**Writing – review & editing:** Rohola Shirzadi, Saminnaz Kazemi, Seyed Hossein Mirlohi.
